# Biomedical Data Mining and Machine Learning for Disease Diagnosis and Health Informatics

**DOI:** 10.3390/bioengineering11040364

**Published:** 2024-04-11

**Authors:** Yunfeng Wu, Meihong Wu

**Affiliations:** School of Informatics, Xiamen University, 422 Si Ming South Road, Xiamen 361005, China; wmh@xmu.edu.cn

Powered by biomedical data mining and machine learning technologies, smart healthcare uses cutting-edge medical innovative tools to facilitate the development of sophisticated decision support systems for disease diagnosis and health informatics. By analyzing large medical datasets, biomedical data mining and machine learning technologies enable the smart healthcare systems to identify subtle patterns and correlations that may be missed by human observation, which helps predict the likelihood of disease progression based on previous medical history and provide early intervention and personalized treatment plans. The smart healthcare systems can also analyze the real-time data of physiological conditions of patients from different sources, such as electronic health records, electrophysiological signals, imaging, and genomic data, to provide clinicians with accurate treatment recommendations, drug interactions, and potential adverse effects.

Since the end of 2019, the COVID-19 pandemic has made a significantly impact on global public health. The mutation and highly contagious nature of the SARS-CoV-2 virus caused rapid spread of interpersonal COVID-19 infections all over the world, resulting in widespread economic recessions and disruptions of social activities. During the early stages of the COVID-19 pandemic, hospitals and clinics were inundated with patients, leading to shortages of essential medical supplies. This situation calls for robust contingency plans and stockpiles of critical resources to respond effectively to public health emergencies.

The signs and symptoms of COVID-19 include dry cough, fever, pneumonia, lymphopenia, fatigue, myalgia, dyspnea, sneezing, and chills. The early detection and diagnosis of COVID-19 played pivotal roles in monitoring the pandemic’s progression and informing on public health circumstances. Timely identification of infected cases enabled prompt implementation of some control strategies, such as isolation, social distancing, contact tracing, and quarantining, to reduce viral transmission within communities. When the infected individuals were identified, immediate isolation was an effective way to prevent further infections in the broader community. Timely diagnosis ensures that infected individuals receive appropriate medical care for early intervention to reduce the risk of severe symptoms. In this case, the limited healthcare resources, such as hospital beds, personal protective equipment, ventilators, and intensive care unit (ICU) admissions, could be allocated more effectively. Early detection and diagnosis of COVID-19 can allow for the identification of high-risk groups, for example, the elderly or those with underlying health conditions, who could be more susceptible to severe COVID-19 complications. This ensures the appropriate targeted interventions and preventive strategies are considered to protect the high-risk population.

Several COVID-19 detection techniques have been developed and regularly used in practice [1,2,3]. The prevailing detection methods include reverse transcription-polymerase chain reaction (RT-PCR), reverse transcriptase-loop-mediated amplification (RT-LAMP), immunoassay antibody detection for point-of-care testing (POCT), biosensor-based detection, radiography imaging screening, and acoustic detection based on cough and breathing sounds [1,2,3].

The RT-PCR technique is a specialized form of PCR specifically designed for the detection of RNA molecules [1]. Such a technique is reliable and able to handle a high volume of samples simultaneously and produce results within a few hours. RT-PCR became the benchmark detection method for SARS-CoV-2; because it directly measures the presence of viral genetic material, RT-PCR could provide higher sensitivity and accuracy than alternative methods detecting viral antigens or antibodies. The RT-PCR process mainly involves two consecutive reactions: first, the RNA is converted into complementary DNA by reverse transcriptase; second, the polymerase chain reaction amplifies the complementary DNA samples using gene-specific primers, illustrating the presence of the target gene through the use of fluorescently labeled TaqMan hydrolysis probes. Although RT-PCR has been extensively used as the gold standard diagnostic method for the diagnosis of COVID-19, concerns are still being raised for tackling its potential limitations and maintaining confidence in the testing results [1].

The RT-LAMP technique is an alternative COVID-19 diagnosis method that could meet the demand for rapid, robust, and highly sensitive testing [1]. The RT-LAMP reaction requires four to six primers and occurs at the temperature range of 60–65 °C, ensuring its testing accuracy and efficiency. With a single-step reverse transcription reaction of unpurified RNA, the LAMP reaction could significantly accelerate sample processing and reduce the overall reaction time for viral detection (less than approximately 2 h compared to the RT-PCR testing time) [1].Both RT-PCR and RT-LAMP can be used for viral infection chain tracing, and novel biomedical data mining methods would work effectively to identify the possible spread of COVID-19 infections in communities.

COVID-19 could also be identified through antigen and antibody tests, which are also referred to as POCT detection [2]. According to immunology principles, the presence of the SARS-CoV-2 antigen indicates an active viral infection. But antigen density depends on individual immune reactions. In screening practice, the antibody-based detection of SARS-CoV-2 is more sensitive than antigen-based detection. The detection of immunoglobulins M (IgM) and G (IgG) are most commonly used for screening of SARS-CoV-2 antibodies. During the viral infection, IgG concentrations in the serum significantly exceed those of IgM. Therefore, immunoassays, such as enzyme-linked immunosorbent assays (ELISAs) and lateral immunoassays in the form of colloidal gold or immunofluorescence, can be effectively utilized for SARS-CoV-2 detection [2].

Radiology examinations such as chest X-ray or lung computed tomography are also effective methods used for diagnosis of COVID-19 [3]. The CT imaging features associated with COVID-19 infection include ground-glass opacities (the presence of hazy, non-specific areas of increased lung density), patchy and peripheral regions of the lungs, and asymmetric opacities areas between the left and right lung. Manual screening of CT images is time consuming, and the recently developed artificial intelligence and machine learning methods have been effectively utilized for identification of COVID-19 signs and monitoring the progression of the disease. The topics of advanced image analysis methods of X-ray or CT images falls into the scope of this Special Issue.

Recent studies have provided some evidence that the respiratory patterns such as breathing, coughs, or vocal sounds of patients infected with COVID-19 are altered and distinct from those of healthy individuals [3,4]. Therefore, the respiratory signal processing and auscultation data analysis techniques can be effectively applied to distinguish the pathological patterns of speech recordings acquired by stethoscope or acoustic sensors. Advanced machine learning algorithms and deep learning models are promising to identify the subtle changes in breathing and cough patterns related to viral infection. This Special Issue plans to feature the latest research works on pathological speech pattern analysis related to respiratory diseases and dysphonia.

Surveys have shown that many COVID-19 survivors continue to suffer from long COVID even after initial recovery from SARS-CoV-2 infection [4,5]. Long COVID refers to a multi-system disease with symptoms that may last several years or even a lifetime [4]. In general, symptoms of long COVID include fatigue, muscle pain, palpitations, cognitive impairment, dyspnoea, anxiety, chest pain, and arthralgia [5]. The persistence and diversity of these long-term neo-coronavirus symptoms reflect chronic damage to multiple organ systems, placing significant burden on the quality of life for COVID-19 survivors.

The pathophysiology of long COVID involves multiple aspects [4]. In the respiratory system, SARS-CoV-2 initially infects the alveolar epithelial cells, potentially triggering a chronic inflammatory response leading to the sustained production of inflammatory cytokines and reactive oxygen species. This chronic inflammation may cause fibrotic changes in lung tissue, impairing lung function. A proportion of COVID-19 survivors exhibit signs of respiratory distress, lung damage, breathing difficulties, or reduced exercise capacity. To address the respiratory sequelae of long COVID, smart healthcare systems could integrate multiple diagnostic tools and technologies, including advanced sensors for lung function tests; high-resolution lung CT imaging to assess fibrosis and inflammation; wearable devices like smartwatches for monitoring vital signs; and machine learning models to analyze extensive medical data for disease pattern recognition, prognosis prediction, and personalized treatment suggestions. Such smart healthcare solutions could provide comprehensive assessment and monitoring, aiding in improving treatment outcomes and quality of life for COVID-19 survivors.

Long COVID can also lead to some cardiovascular disorders [4]. The ACE2 receptors on cardiomyocytes provide a pathway for SARS-CoV-2 infection, potentially leading to myocarditis and cell death. Chronic inflammation and cellular damage may result in cardiac fibrosis, increasing the risk of arrhythmias and coagulation disorders. Autonomic nervous system inflammation can lead to postural orthostatic tachycardia syndrome (POTS). Studies have indicated that COVID-19 patients may experience persistent myocardial inflammation and cardiac damage, including those not hospitalized. The monitoring of cardiovascular sequelae could be implemented with continuous heart activity tracking with smart echocardiogram devices, along with arrhythmia detection by using high-precision sensors. Advanced imaging techniques like echocardiography, powered by deep learning algorithms, could be used to analyze images and identify signs of myocarditis or other cardiac damage.

Dysfunction of the central nervous system (CNS) is another characteristic of long COVID-19 [6]. Chronic neuroinflammation may cause neurodegenerative diseases, because SARS-CoV-2 can cross the blood–brain barrier, further promoting neuroinflammation in brain tissue. Such an inflammatory and hypercoagulable state could increase the risk of thrombotic events and cause autonomic nervous system dysfunction. Dysfunction of the CNS in the brain may result in long-term cognitive impairments and other neuropsychiatric symptoms like chronic malaise, sleep disturbances, loss of taste and smell, and post-traumatic stress disorder. Machine learning algorithms can assist in the analysis of magnetic resonance imaging or CT scans to identify the subtle abnormal changes and potential inflammatory regions in the brain structure. Long-term EEG monitoring could be used to detect abnormal neurological patterns for comprehensive assessment and personalized treatment to improve prognosis and quality of life.

In addition, long COVID can also affect other organs such as the kidneys, pancreas, and gastrointestinal tract [4]. Excessive inflammation in the renal tissue may lead to glomerulosclerosis and reduced kidney function. Pancreatitis and systemic inflammation can impair pancreatic function, while changes in the gastrointestinal system may result in microbiome imbalances and damage to gastrointestinal integrity. With smart medical diagnostic tools, physicians can comprehensively assess and manage the renal, pancreatic, and gastrointestinal sequelae in long COVID patients, offering more precise medical care.

This Special Issue is dedicated to leveraging biomedical data mining and machine learning technologies to delve into the pathophysiological mechanisms of this multisystem disease, as well as the long-term repercussions of COVID-19. These advanced technologies can assist in the precise monitoring and prediction of the clinical features of long COVID and effectively discover key clinical factors, laying a solid foundation for the development of scientifically rigorous and multidisciplinary integration of treatment plans. By integrating innovative biomedical data mining and machine learning technologies, smart healthcare systems can provide more accurate and efficient diagnostic solutions [7] and treatment opportunities [8], aiming towards the purpose of significantly improving overall healthcare quality and rehabilitation outcomes [9]. In this Special Issue, we strive to highlight the recent development of biomedical data mining and machine learning technologies for the diagnosis of infectious diseases and chronic diseases; the topics also cover the theoretical advances and practical applications of deep learning neural network architectures for physiological signal measurement and data analysis [10].
